# Fracture of an osteochondroma treated successfully with total excision: two case reports

**DOI:** 10.4076/1757-1626-2-8062

**Published:** 2009-08-07

**Authors:** Ozkan Kose, Abdullah Ertas, Mustafa Celiktas, Bulent Kisin

**Affiliations:** Orthopaedics and Traumatology Clinic, Diyarbakir Education and Research HospitalDiyarbakirTurkey

## Abstract

Fracture of an osteochondroma is a rare complication. We report two cases of fractured osteochondroma in two children that were treated successfully with total excision.

## Case presentations

### Case report 1

**Figure 1.**
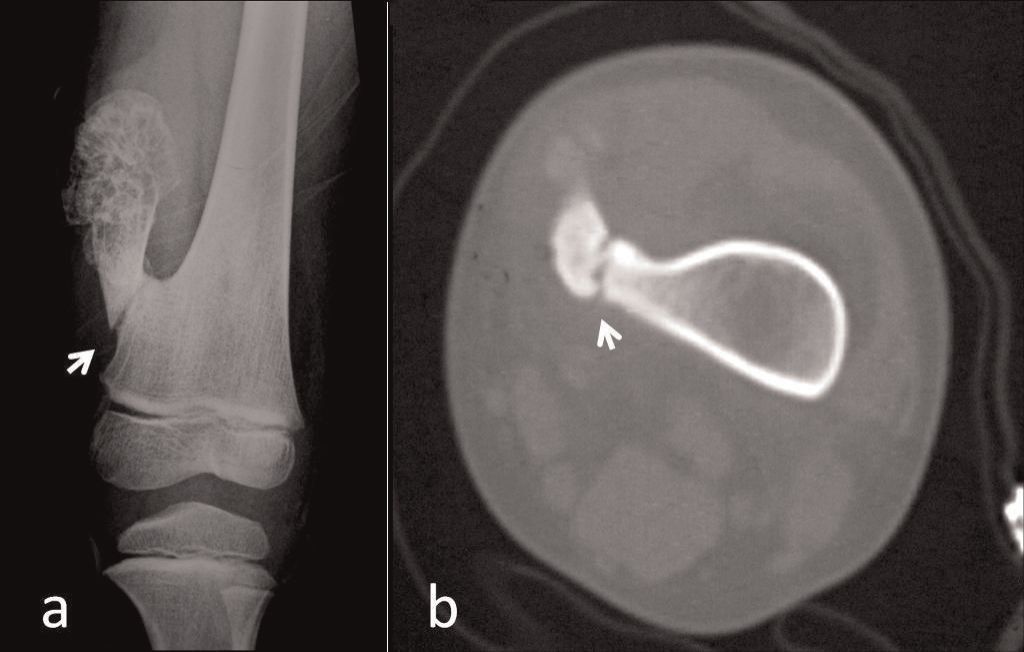
**(a)** Antero-posterior plain radiograph of the femur. **(b)** Axial CT scan of the femur through the fracture line. White arrows show the fracture.

**Figure 2.**
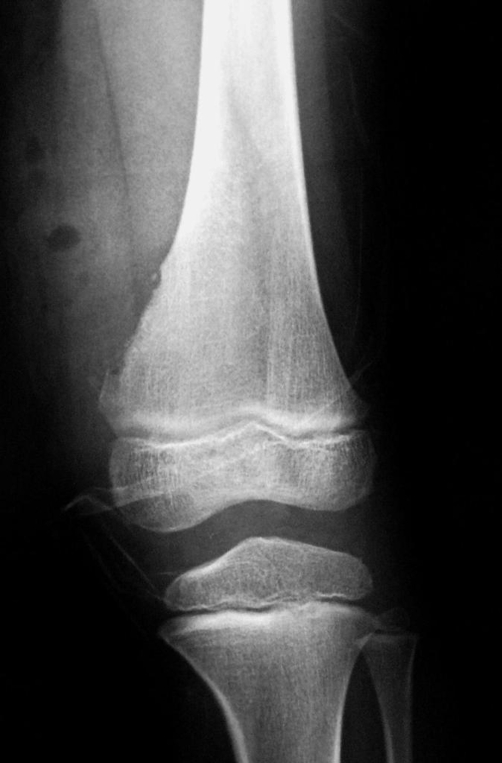
Antero-posterior plain radiographs of the femur immediately after the resection.

A 6-year-old, Caucasian Turkish boy presented to the emergency department with pain in his left thigh after falling down the stairs. On physical examination, there was slight oedema over the distal and medial aspect of his thigh and tenderness with palpation. Knee and hip range of movements were both painful and restricted. Neurovascular examination revealed no abnormality. His past medical history was unremarkable. Distal femoral fracture was initially suspected. Direct radiography demonstrated a fracture through the stalk of a pedunculated osteochondroma originating from adductor tubercle of the femur (Figure 1). Further detailed questioning about the previous symptoms revealed intermittent pain over the osteochondroma particularly after strenuous physical activity. Total excision of the osteochondroma was performed (Figure 2). The diagnosis was also confirmed with histopathology. The post-operative period was uneventful and the patient has returned to his previous level of activity within three weeks.

### Case report 2

**Figure 3.**
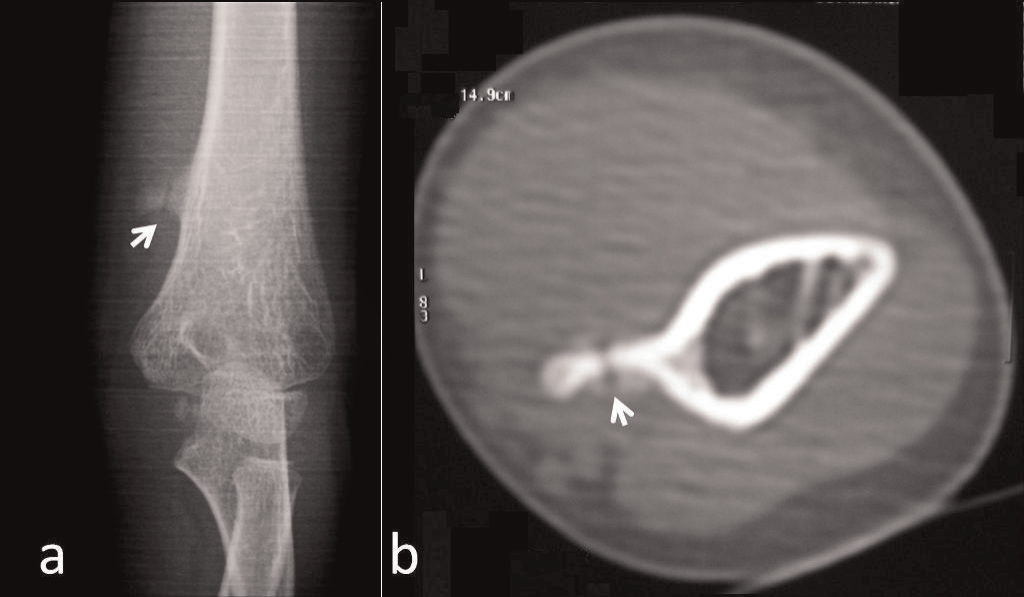
**(a)** Oblique plain radiograph of the humerus. **(b)** Axial CT scan of the humerus through the fracture line. White arrows show the fracture.

An 8-year-old, Caucasian Turkish girl presented to the outpatient orthopaedics clinic with pain in her left arm after her younger sister hit with a stick, a week ago. On physical examination, there was localized tenderness over distal and medial aspect of her arm with palpation. Shoulder and elbow range of movements were normal. Neurovascular examination revealed no abnormality. Her past medical history was unremarkable. Soft tissue injury was initially suspected. Direct radiography demonstrated a fracture of an osteochondroma on the distal medial metaphyseal humerus (Figure 3). She denied any other related symptoms previously. Total excision of the osteochondroma was performed. The post-operative period was uneventful and the patient has returned to his previous level of activity within four weeks.

## Discussion

Osteochondroma is the most common benign tumor of bone that constitutes 10-15 % of all bone tumors [1]. They are considered to be developmental lesions rather than true neoplasms. Osteochondromas are usually asymptomatic and detected as an incidental finding on radiography [2]. However, symptoms may occur in patients in whom the osteochondroma is associated with complications. Various complications of osteochondroma have been described in the relevant literature such as fracture, vascular compromise, entrapment neuropathy, bursa formation, malignant transformation and muscle impingement [1]. Fracture through the stalk of a pedunculated osteochondroma is a rare complication. A few numbers of cases have been reported up to date [2-5]. The treatment of this fracture is controversial. Some authors suggest observation, but others prefer surgical excision. Surgical excision provides faster recovery and return to normal activity [6]. This is also true for the presented case, the patient recovered within three weeks of duration. Immediate total resection is an effective treatment strategy providing rapid recovery and cure for this uncommon complication.

In conclusion, fracture may be the first symptom of an osteochondroma and we recommend immediate surgical excision for this rare complication.

## References

[bib-001] Murphey MD, Choi JJ, Kransdorf MJ, Flemming DJ, Gannon FH (2000). Imaging of osteochondroma: variants and complications with radiologic-pathologic correlation. Radiographics.

[bib-002] Davids JR, Glancy GL, Eilert RE (1991). Fracture through the stalk of pedunculated osteochondromas: a report of three cases. Clin Orthop.

[bib-003] Prakash U, Court-Brown CM (1996). Fracture through an osteochondroma. Injury.

[bib-004] Tanigawa N, Kariya S, Kojima H, Komemushi A, Fujii H, Sawada S (2007). Lower limb ischaemia caused by fractured osteochondroma of the femur. Br J Radiol.

[bib-005] Alonso-Torres A, Bernabéu D, López-Barea F, Martín-Hervás C, González-López JM (2004). Growth and fracture of an osteochondroma in an adult patient. Eur Radiol.

[bib-006] Carpintero P, León F, Zafra M, Montero M, Berral FJ (2003). Fractures of osteochondroma during physical exercise. Am J Sports Med.

